# Natural Born Detourers Modern Utility Dog Breeds Show Ancestry‐Based Superiority in Social Learning Capacity in a Detour Task

**DOI:** 10.1111/eva.70151

**Published:** 2025-08-27

**Authors:** Péter Pongrácz, Petra Dobos

**Affiliations:** ^1^ Department of Ethology ELTE Eötvös Loránd University Budapest Hungary

**Keywords:** detour task, dog breeds, genetic relatedness, social learning

## Abstract

Behaviour has crucial importance in dogs' adaptation to the anthropogenic environment. Functional breed selection, a relatively recent evolutionary event, resulted in strong differences regarding dogs' capacity for observational learning from humans. However, genetic distance among dog breeds has thus far not been connected to their social learning performance. Here we show first evidence that ancestry‐based clustering of dog breeds can result in biologically relevant phenotypic differences in their capacity to learn from humans. We analysed a large database of spatial problem‐solving (detour) tests, where a representative sample (*N* = 174) of cooperative and independent working dogs were sorted into 8 ancestry groups based on a genetic cladogram. We analysed whether ancestry would affect individual and social learning‐based spatial problem‐solving of dog breeds. Our results showed that ancestry groups with today's utility dog breeds performed this task best. Social learning was also prevalent in the ancestry group that collects English herding breeds and sight hounds as well—showing that genetically closely related cooperative and independent working dog breeds can possess similar sociocognitive traits. These results strengthen the notion that the behaviour of dog breeds can provide ecologically valid research opportunities both for proximate and ultimate evolutionary events.

## 1 Introduction

When Morrill et al. (2022) published their large‐scale research on genetically determined phenotypes of dogs in *Science*, many readers interpreted their findings as proof of negligible‐to‐no breed‐specific inherited behaviours (see for a review: Udell 2022). At the same time, it is also emphasized that dogs' behaviour (that apparently shows differences among the breeds) is most probably influenced by environmental effects (e.g., Heurlin et al. 2024). The vivid interest towards, and discussion about the results of Morrill et al. (2022) signifies that dog breeds represent much more than variable, “pretty outfits” that individuals of the same species, *Canis familiaris* , are wrapped into. The traditional description of dog breeds called “breed standards”, (e.g., Zapata and Zapata 2024) and the vast practical knowledge that was collected about the utilization of many dog breeds as sporting and working animals, equally suggests that the breeds not only look different, but also show markedly characteristic behaviours (Coppinger and Coppinger 2024). The often heated scientific and societal debate about the extent of signature breed‐related behaviours can have its advocates, for example when the goal is to select suitable breeds for a given task (e.g., livestock guarding dogs against wolves, Kinka and Young 2019). However, there are just as fierce opponents of this idea, for example when there are legislative bans against the so‐called “dangerous” dog breeds (for cons, see for example Collier 2006; Schalke et al. 2008).

Although Morrill et al. (2022) convincingly showed that there is no evidence for single breed‐specific behavioural genotypes, we should not forget another important finding of these authors: they showed that there is a well detectable, overarching trait, “biddability”, which shows excellent match with functional breed selection. Those breeds that were selected for highly cooperative tasks with their handlers, show higher genetic predisposition for such behavioural features that belong to being “biddable”, unlike those breeds that were selected for performing their job mainly without constant human cueing. This warrants the researcher that when the goal is to find ecologically valid explanations for the apparently different behaviours in various dog breeds, we should always look for wider, biologically relevant grouping variables, which can reveal how the given behavioural phenotype in question has been selected (see for a review, Pongrácz and Dobos 2025).

So far, the most often encountered research method for behavioural comparisons among dog breeds is convenience sampling, where researchers select their subjects based on the most available breeds at a given place and time (e.g., Salamon et al. 2025). If this is accomplished with an appropriately large sample size, this method can serve as a useful tool for the initial detection and description of behavioural phenomena (e.g., looking for between‐breed differences of human‐directed aggression in Finland, Mikkola et al. 2021). However, if researchers intend to test a priori research hypotheses, the purposefully, and representatively chosen breeds, or breed groups, offer a biologically more relevant option (Pongrácz and Dobos 2025). One of these overarching grouping methods is the already mentioned functional breed selection, where the researchers hypothesize that dog breeds that were selected for either working in a cooperative manner with their handler, or mainly independent of human feedback, would behave differently when they interact with humans (e.g., Gácsi et al. 2009; Pongrácz et al. 2020). The other, widely utilized approach for hypothesis‐driven behavioural breed comparisons is based on the genetic relatedness of dog breeds (Dutrow et al. 2022). This idea capitalizes on the common origin of all the extant dogs (Vilà and Leonard 2007). According to this, subsequent diversification from the common canine ancestor not only resulted in the emergence of increasingly “derived” anatomical features (compared to the “basal”, or “wolf‐like” look); but a similar process also caused an ancestry‐based behavioural divergence among the fundamental types of dogs (Dutrow et al. 2022). By using large‐scale molecular genetic methods, it was possible to create intricate “cladograms”, which comprise hundreds of dog breeds that cluster into several clades of more closely related breeds (e.g., Parker et al. 2017). These clades show quantifiable genetic distances from the common ancestral node, thus by using these cladograms as a proxy for genetic relatedness between clusters of dog breeds, researchers can conduct hypothesis‐driven behavioural comparisons among the breeds (Turcsán et al. 2011).

Not surprisingly, much of the genetic relatedness‐based research was aimed at comparisons between the so‐called “ancient” (also called as “primitive” or “basal”) and “modern” dog breeds (for a review, see Pongrácz and Dobos 2025). These investigations build on the behavioural differences found between socialized wolves and dogs (e.g., Miklósi et al. 2003; Topál et al. 2005), by assuming that breeds (or breed groups) falling closer to the wolf‐like ancestor, would show more wolf‐like behavioural phenotypes (e.g., Vaysse et al. 2011). Such studies, among others, were aimed at the ability of visual contact seeking (Konno et al. 2016); testing for sociability and reactivity (Hansen Wheat et al. 2019); and prevalence of problem‐behaviours, such as low trainability, and tendency to roam and escape (Smith et al. 2017). However, ancestry‐based breed comparisons appear in applied ethological research as well. Hammond et al. (2022) compared the behavioural traits of “dangerous breeds” that were put under legislative ban, with the same traits in non‐legislated breeds, in which they based their breed‐choice on the cladogram of Parker et al. (2017). Using the same genetic clustering, Kolkmeyer et al. (2024) compared the effect of spaying/neutering on aggressive behaviours in the two most distant genetic clades: the “primitive” dogs (such as the Husky and Chow Chow) and the most “derived” dog breeds (such as the Boxers and Mastiffs).

One of the biggest challenges of using ancestry‐based clusters in behavioural comparisons of dog breeds is the confounding effect of functional breed selection (Pongrácz and Dobos 2025), because in many genetic clades we can find both cooperative and independent working dog breeds. For example, according to Parker et al. (2017), independently working sight hounds such as Whippets and Greyhounds, are most closely related to the cooperative worker herding dog breeds (Border Collies, Australian Shepherds). There are also studies, which based their methods on the genetic relatedness between the breeds in their sample but could not really account for the work‐related selection as confounder (e.g., Dorey et al. 2009; Gnanadesikan et al. 2020).

Our goal in this investigation was to test whether relatedness‐based clustering of a wide selection of working dog breeds would result in ancestry‐related differences in a spatial‐cognitive task, where recently we found a robust effect of functional breed selection on dogs' behaviour (Dobos and Pongrácz 2023, 2024). By using the detour paradigm, it was shown that both cooperative and independent breeds faced similar difficulties when they attempted to detour on their own. However, cooperative breeds effectively learned from a human demonstrator how to detour easily, while the independent breeds did not benefit from observational learning. In our sample we had a wide selection of dog breeds from the various clades described by Parker et al. (2017), however, to our best knowledge, so far, no ancestry‐based analysis was conducted on dogs' social learning capacity or performance in spatial problem solving. As the detour task includes both individual problem solving and social interaction with humans, we wanted to know whether the response of the various ancestry groups would be influenced by the potentially higher individual problem‐solving skills of the “basal breeds” (higher persistence, lower dependence on humans, Sommese et al. 2019); or the more modern clades would perform better, where the dog breeds were likely directly selected for interacting with humans (Nagasawa et al. 2017) (i.e., paying attention to a demonstrator). As training and keeping conditions of the dogs can represent a strong confounder effect (Azadian and Protopopova 2025), we also took into consideration these factors during the analysis.

## 2 Methods

This research is based on the unified database of purebred dogs, who were tested in three previous studies (Dobos and Pongrácz 2023, 2024; Pongrácz and Veres 2025). Each study was based on the detour paradigm, in which dogs had to go behind a transparent, V‐shaped fence to reach the reward, either with the help of observing a human demonstrator's action, or on their own, without demonstration.

### 2.1 Subjects

The subjects of this study were companion dogs (*N* = 169), who participated in the original tests with their owner. The participants were recruited via advertisements in social media. The selection criteria for this study were that the dogs had to be purebred, and they had to be tested with the V‐shaped fence in the detour task. Thus, we included the data of *N* = 77 dogs from Dobos and Pongrácz (2023); *N* = 69 dogs from Dobos and Pongrácz (2024); and *N* = 23 dogs from Pongrácz and Veres (2025). Throughout the sample collection, we paid utmost attention to include the possible widest selection of dog breeds, which represented either the independent, or the cooperative working functional categories (Dobos and Pongrácz 2023, 2024). Dogs were at least 1 year old, and we originally also recorded their keeping conditions (indoor‐only or indoor‐outdoor); and their highest level of training (none; only at home; single course at dog school; regular dog school; private trainer; special training). Signalment data of the subjects can be found in the raw data file in the Supporting Information.

### 2.2 Clustering of the Dog Breeds According to the Multi‐Breed Clades Defined by Parker et al. (2017)

In our sample we had an unequal number of dogs that belong to the individual 23 multi‐breed clades defined by Parker et al. (2017). Therefore, for the analysis, we performed further clustering of the most closely related clades, which sat on the nearest neighboring “branches of the cladogram”. This resulted in 11 “Ancestry groups”. From these groups, eventually eight had a high enough number of subjects to analyse (Table 1).

**TABLE 1:** List of the participating dog breeds in this study. “Ancestry groups” of dogs were created by clustering of the most closely related original clades defined by Parker et al. (2017).

Clade (Parker et al. 2017)	Breeds (and *N*s) included to this study from the clade	Ancestry group (created for present study)	Total number of dogs in the ancestry group
A	*Siberian husky (N = 1)* *Shiba inu (N = 2)* *Akita inu (N = 1)* *Shar‐pei (N = 1)* *Samoyed (N = 2)* *Yakutian laika (N = 5)*	Group 1 “Basal dogs”	12
B	—		
C	—		
D	—	Group 2	N/A
E	—		
F	—		
G	Mudi (*N* = 8) Puli (*N* = 12) Pumi (*N* = 4)	Group 3 “Hungarian herding dogs”	14
H	—	Group 4	N/A
I	—	Group 5 “Terriers”	16
J	—		
K	*Miniature Pinscher (N = 1)*		
L	*Bedlington terrier (N = 2)* *Cairn terrier (N = 1)* *Fox terrier (N = 4)* *Irish terrier (N = 2)* *Jack Russell terrier (N = 5)* *Westie (1)*		
M	German shepherd dog (*N* = 3) *Hovawart* ^a^ *(N = 6)*	Group 6 “German Shepherd Dog”	9
N	—	Group 7	N/A
O	*Dachshund (N = 14)* *Basset Hound (N = 3)* *Polish hound (N = 1)* *Transylvanian hound (N = 8)*	Group 8 “Hounds, gundogs”	58
P	English Cocker Spaniel (*N* = 7)		
Q	Golden Retriever (*N* = 4) Labrador Retriever (*N* = 8)		
R	Irish setter (*N* = 3) Lagotto Romagnolo (*N* = 4) Vizsla (*N* = 5) Weimaraner (*N* = 1)		
S	Bouvier des Flandres (*N* = 2) Briard (*N* = 5) Tervueren (*N* = 1)	Group 9 “Briard and Belgian sheepdogs”	8
T	Australian shepherd (*N* = 8) *Borzoi (N = 2)* Border Collie (*N* = 18) Cardigan Welsh Corgi (*N* = 1) Collie (*N* = 2) *Galgo (N = 2)* *Greyhound (N = 1)* Sheltie (*N* = 3) *Whippet (N = 5)*	Group 10 “Sight hounds, English herding dogs”	42
U	Rottweiler (*N* = 2)	Group 11 “Bull‐type dogs”	10
V	—		
W	Boxer (*N* = 1) *Boston terrier (N = 1)* *Cane Corso (N = 1)* *English bulldog (N = 1)* *Bullterrier (N = 2)* *Pitbull Terrier (N = 2)*		

*Note:* In the case of those ancestry groups that were included to this study, we also added the reference name as we mention these across the text. The ascending numbers of the ancestry groups represent the increasing genetic distance from the common ancestor. N/A = we did not have any, or enough dogs for analysis. Italicized dog breed names represent “independent working dogs”, and breed names in normal print mean “cooperative breeds”.

^a^ The original Parker et al. (2017) study did not include the Hovawarts, it was more recently concluded that this breed has close connections with various old German and Italian herding dogs as well as with the German Shepherd Dog and other related breeds (Ulbricht 2023).

### 2.3 Testing Methods

**FIGURE 1:**
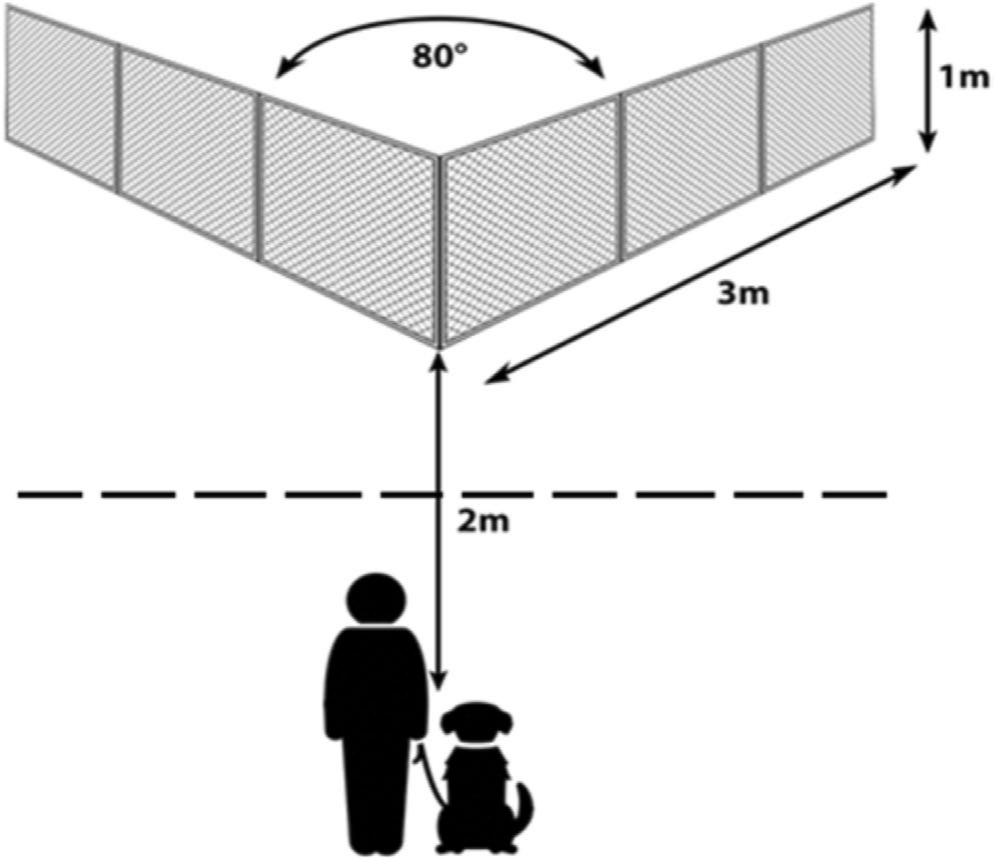
Schematic drawing of the experimental device and the position of the dog at the starting point with the owner. The reward was always placed to the inner corner of the V‐shaped fence, where the dog was able to obtain it only after making a successful detour along one of the wings of the fence. In the case of demonstration, the experimenter started to walk towards the fence also from the starting point, where she returned after making a detour and leaving the reward behind the fence.

The detailed protocols can be found in the original publications; however, we provide a brief resume for convenience. Each dog was tested in three consecutive trials in the detour task, at a flat, grassy location at the campus of the Eötvös Loránd University, Budapest, Hungary. The obstacle was a transparent, V‐shaped wire mesh fence with light metal frame, fastened firmly and tightly to the ground with protruding metal pegs. Each wing of the fence was 3 m long and 1 m high, and the two wings joined with an 80° intersecting angle. The starting point was 2 m from the corner of the fence, with the target (food or toy, depending on the dog's preference) being placed to the inner corner, behind the fence (Figure 1). During the test, the owner remained at the starting point, where they were allowed to encourage the dog verbally, however, directional signals (visual or verbal) were disallowed. In each trial, dogs had a maximum of 60s to detour the fence and reach the reward. If they could not solve the task, they were assigned with 60s as latency. Besides measuring the latency of reaching the reward (s), we also assigned each trial with a binary “success” score (0 = the dog could not detour the fence in 60 s; 1 = successful detour).

Dogs from three experimental conditions were included to the analysis. Control (without demonstration) (*N* = 48—Dobos and Pongrácz 2023; Pongrácz and Veres 2025); detour demonstration with ostensive speech (*N* = 89—Dobos and Pongrácz 2023, 2024; Pongrácz and Veres 2025), and detour demonstration with neutral speech (*N* = 34—Dobos and Pongrácz 2024).

In the case of the Control condition, dogs had to solve the detour task on their own in each of the three consecutive trials. The reward was placed to the inner corner of the fence by the experimenter (22 years old woman) at the beginning of each trial, who reached over the fence at the corner to do this. In the case of the two demonstration conditions, the experimenter showed the detour to the dogs before Trial 2 and Trial 3. In Trial 1 the dogs had to detour the obstacle without a priori demonstration (like the control condition). While the experimenter demonstrated the task, (by walking around the V‐shaped fence), she visibly carried the reward, then conspicuously placed it to a plate on the ground in the inner corner of the fence. During this time, the dog was kept on leash by the owner at the starting point. In the ostensive speech condition, the experimenter directed the dog's attention to herself by calling the dog's name and using other, ostensive intonated utterances (e.g., “Watch me”). In the neutral speech condition, the experimenter recited a short poem during the demonstration in a non‐ostensive intonation. In each case, the dog was unleashed and encouraged to solve the task after the experimenter had returned to the starting point.

### 2.1 Behavioural Coding

For this analysis, we used the following behavioural variables (see Table 2).

**TABLE 2:** The list and description of behavioural variables used in this study.

Behavioural variable	Unit	Description
Success	Occurrence (0–1)	The dog reaches the reward after performing a successful detour, then it touches/consumes the reward
Reward latency	(s)	The time elapsed between the moment of releasing the dog by the owner at the starting point and the dog's arrival to the reward (i.e., after a successful detour). In the case of an unsuccessful trial, *60s* was assigned
Demonstration duration	(s)	The duration of the Experimenter's demonstration, measured between her departure from the starting point and arrival back to the external apex of the fence
Demonstration looking time	Time percentage %	During demonstration, the dog looks at the demonstrator. The *overall duration of looking* is then divided by the *duration of the demonstration*

### 2.2 Statistical Analysis

A file with all raw data can be accessed in the Data S1 to this article and at Mendeley Data (Pongrácz and Dobos (2025), “Ancestry_detour_dogs”, Mendeley Data, V2, doi: 10.17632/yyg9cw67hk.2). Statistical analysis was performed with the IBM SPSS (version 29) software. Latencies were analysed with Cox regression, success rates with GEE (Generalized Estimating Equations) with binary logistics, and relative duration of watching the demonstrator was analysed with GEE (linear logistics). Detour latency, success, and relative duration of watching the demonstrator (only in Trials 2 and 3) were used as dependent variables. Ancestry group, breed function (cooperative or independent working dogs), type of demonstration, keeping conditions, and training level, were used as fixed factors. In the case of within‐subject comparisons, trial was also added as fixed factor, and subject ID was used as random factor. In the case of the analysis of watching the demonstrator, the dog's success was also added as fixed factor to the model. We also added two‐way interactions to the models, and we performed backward model selection, where we removed the non‐significant interactions one by one. We report the final (simplest) model everywhere, by keeping all the main effects (whether they were significant or not). Level of significance was everywhere (*α* = 0.05).

## 3 Results

**FIGURE 2:**
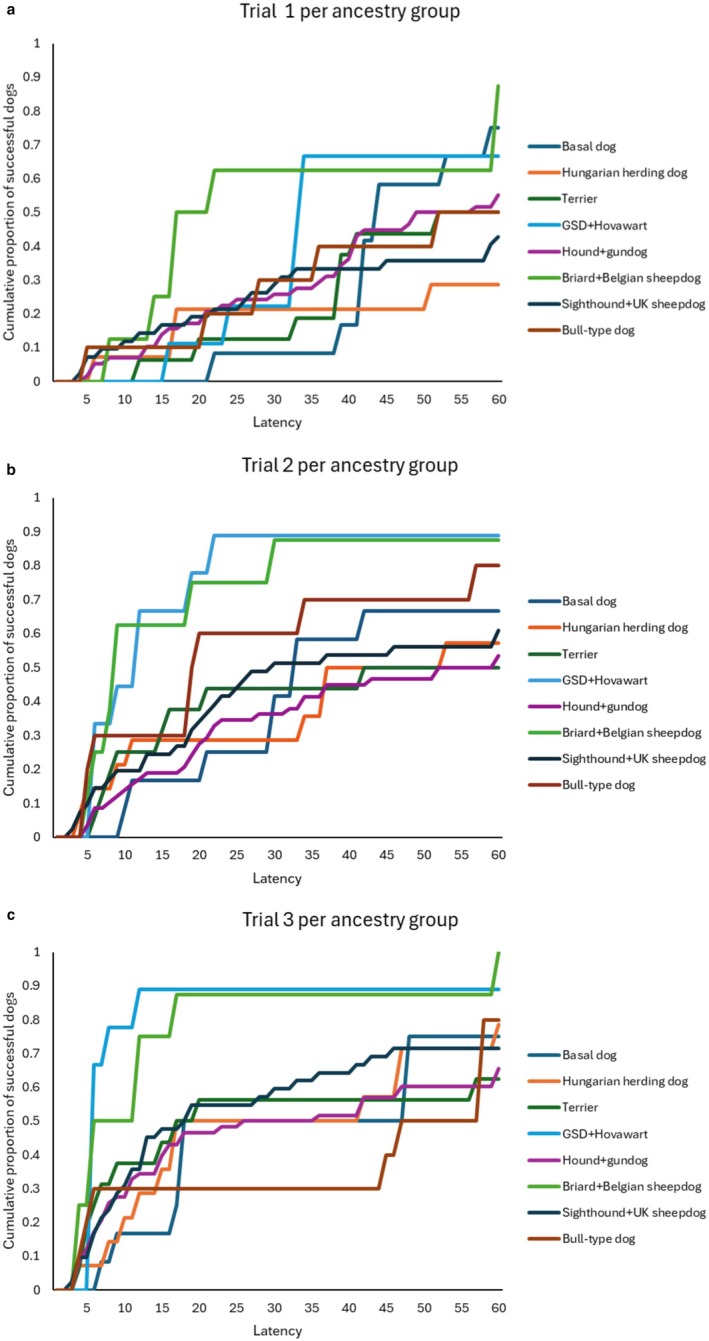
Detour latencies in Trial 1. There is no significant difference between the ancestry groups. (b) Detour latencies in Trial 2. Group 6 (GSD + Hovawart) and Group 9 (Briard + Belgian sheepdogs) made the fastest detour. (c) Detour latencies in Trial 3. Group 6 (GSD + Hovawart) and Group 9 (Briard + Belgian sheepdogs) made the fastest detour, the other groups do not differ from each other. GSD, German Shepherd Dog.

Based on the full dataset, we analysed whether the detour latencies differ in the consecutive trials (Table 3). Ancestry group, breed function, type of demonstration, keeping and training were the fixed factors. None of the factors had significant association with the detour latency in Trial 1 (Figure 2). However, ancestry group had a strong significant association with the latencies in Trials 2–3. Type of demonstration, breed function, keeping and training did not associate significantly with detour latencies in Trial 2 and 3.

**TABLE 3:** Results of the Cox regression analysis, when we tested whether the ancestry group, breed function, type of demonstration, keeping conditions and training level would associate with their detour latencies in the three trials.

Trial	Independent variable	Wald *χ* ^2^	df	*p*
Trial 1	Ancestry group	7.848	7	0.346
	Breed function	0.167	1	0.683
	Demo type	3.872	2	0.144
	Keeping	2.212	1	0.137
	Training	1.281	5	0.258
Trial 2	**Ancestry group**	**24.879**	**7**	**< 0.001**
	Breed function	2.499	1	0.114
	Demo type	0.025	2	0.988
	Keeping	0.100	1	0.752
	Training	0.000	5	0.998
Trial 3	**Ancestry group**	**24.390**	**7**	**< 0.001**
	Breed function	3.290	1	0.070
	Demo type	0.218	2	0.897
	Keeping	0.012	1	0.913
	Training	0.000	5	0.986

*Note:* Significant results are highlighted in bold.

In Trial 2, dogs in ancestry Group 6 (equals with Clade M from Parker et al. (2017) contains *German Shepherd Dogs* and *Hovawarts*) and ancestry Group 9 (Clade S from Parker et al. (2017), contains the *Briard* and *Belgian sheepdogs*) performed the fastest detours, while the other groups did not differ from each other (Figure 2). In Trial 3 Group 6 and Group 9 were again significantly the fastest, while the other groups did not differ from each other (Figure 2).

**FIGURE 3:**
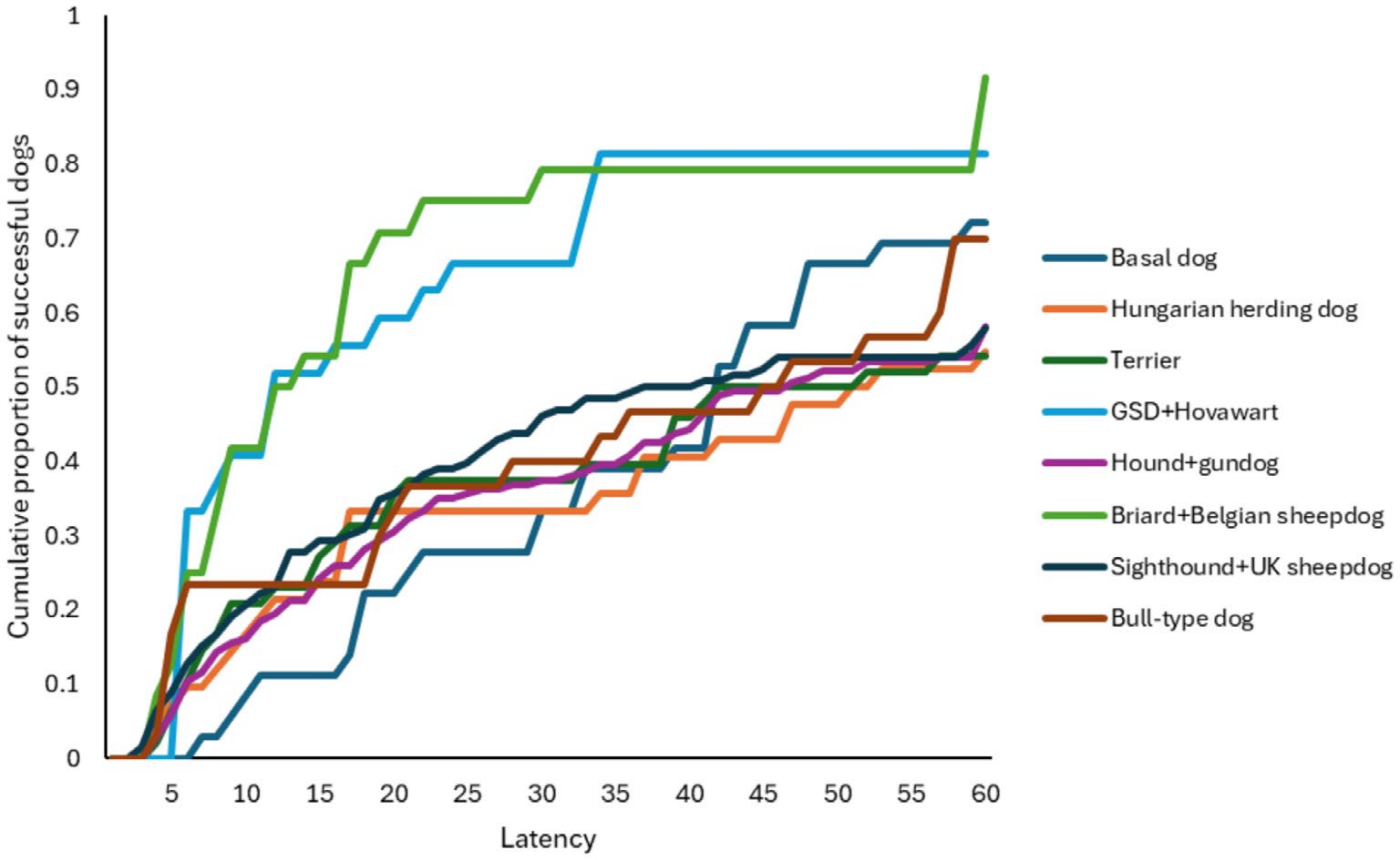
Detour latencies of each ancestry group, all trials together. Dogs in Group 6 (GSD + Hovawart) and Group 9 (Briard + Belgian sheepdogs) performed significantly better than the other groups. GSD = German Shepherd Dog.

**FIGURE 4:**
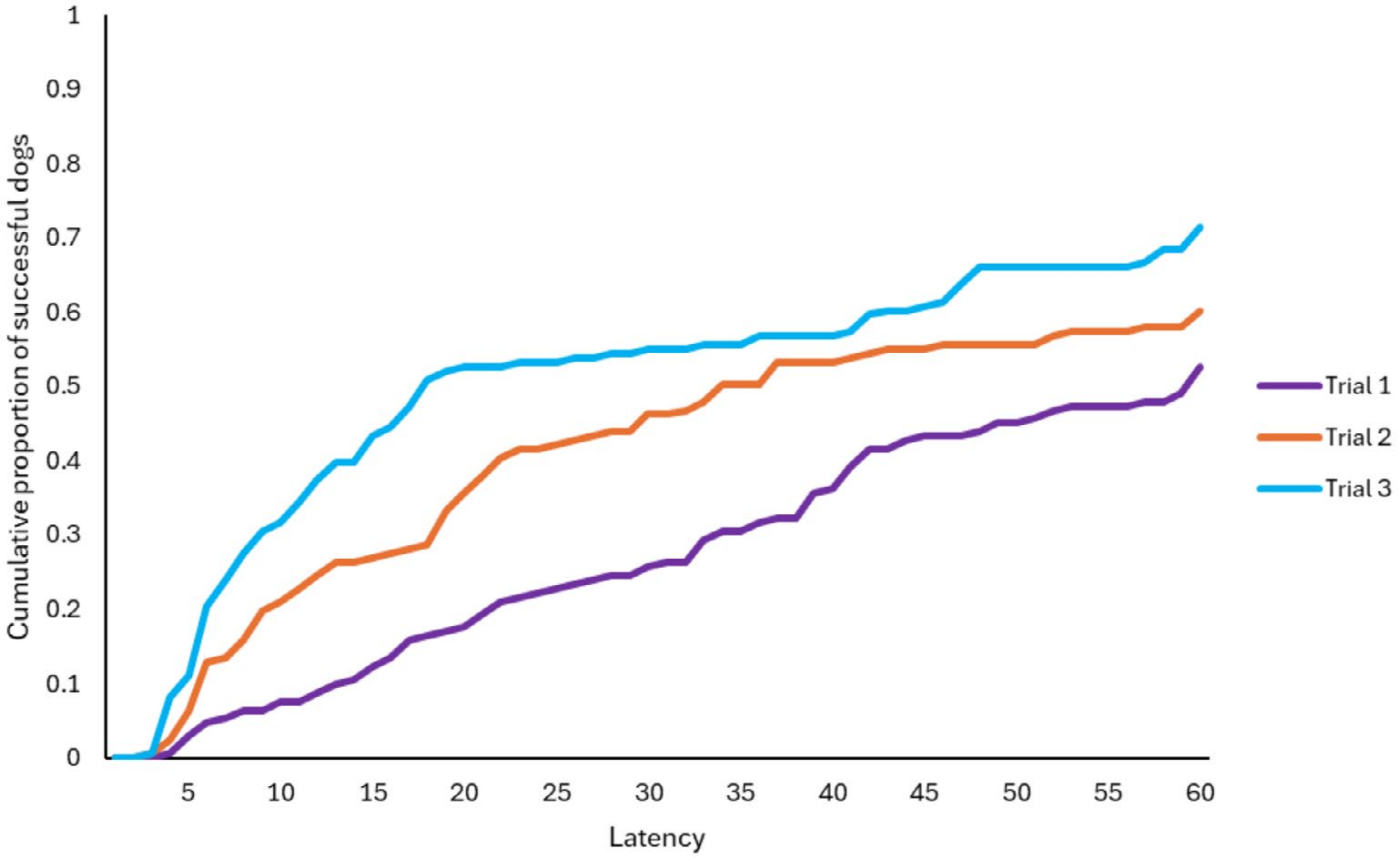
Detour latencies in the three consecutive trials, all groups together. Dogs detoured with significantly shorter latency from trial to trial.

Next, we analysed using the full sample, whether the dogs' detour latencies changed between the trials (within‐subject analysis). Ancestry group (*χ* ^2^_(7)_ = 47.228; *p* < 0.001), breed function (*χ* ^2^_(1)_ = 5.758; *p* < 0.016), and trials had a significant association with detour latency (*χ* ^2^_(2)_ = 29.895; *p* < 0.001). With all the trials together in the model, Group 6 and Group 9 performed the fastest detours (Figure 3). Regarding the trials' effect, dogs' performance became faster from trial to trial (Figure 4). Dogs from cooperative breeds made faster detours than the independently working dogs. The other fixed factors had no significant effect: demonstration (*χ* ^2^_(2)_ = 0.372; *p* = 0.830); keeping (*χ* ^2^_(1)_ = 0.751; *p* = 0.386); training (*χ* ^2^_(5)_ = 0.253; *p* = 0.615).

In the next step we analysed separately whether the individual ancestry groups' detour latencies improved from trial to trial. We must keep in mind that some of the sample sizes were rather low in the case of these analyses (Table 4).

**TABLE 4:** Results of the trial‐by‐trial comparisons in the case of detour latencies in the ancestry groups (Cox regressions).

Ancestry group	*N*	Wald *χ* ^2^	df	*p*
1 (Clades A, B, C/basal breeds)	12	0.558	2	0.756
**3 (Clade G/Hungarian herding dogs)**	**14**	**6.349**	**2**	**0.042**
5 (Clades K, L/terriers)	16	1.741	2	0.419
**6 (Clade M/Hovawart, GSD)**	**9**	**8.217**	**2**	**0.016**
8 (Clades O, P, Q, R/gundogs, hounds)	58	3.729	2	0.155
9 (Clade S/Briard, Belgian sheepdogs)	8	3.794	2	0.150
**10 (Clade T/sight hounds, English herding dogs)**	**42**	**10.294**	**2**	**0.006**
11 (Clades U, V, W/Rottweiler, bull‐type dogs)	10	1.108	2	0.575

*Note:* Significant associations are highlighted with bold typeset.

**FIGURE 5:**
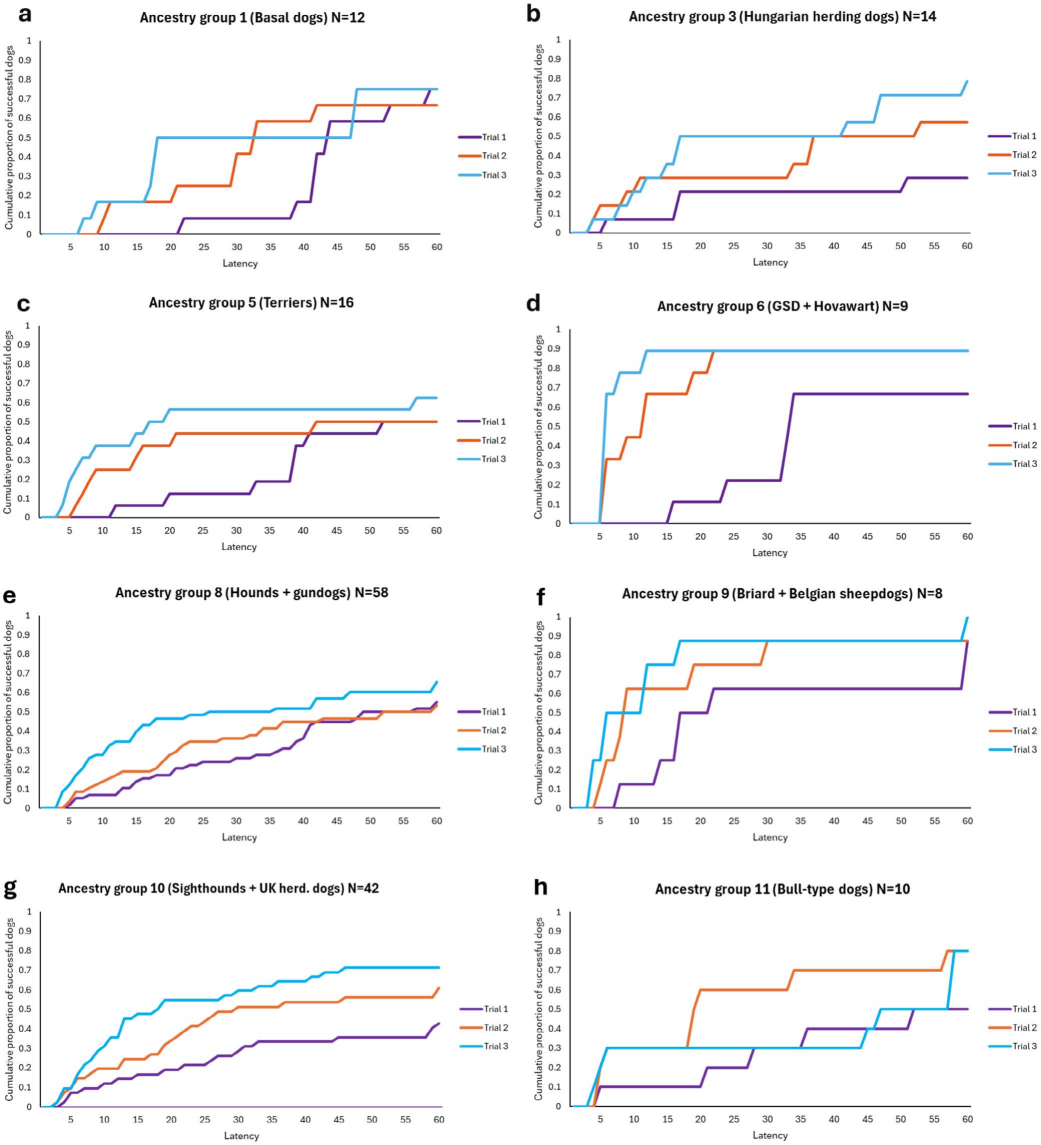
(a–d) The cumulative proportion of successful dogs from ancestry groups 1, 3, 5 and 6 in Trials 1–3. We found significant decrease of latency in Group 3 (Hungarian herding dogs) and Group 6 (German Shepherd Dogs and Hovawarts). (e–h) The cumulative proportion of successful dogs from ancestry groups 8, 9, 10, and 11 in Trials 1–3. We found significant decrease of latency in Group 10 (Sight hounds and English herding dogs).

From among the ancestry groups, we found significant difference between the latencies from trial to trial in Group 3 (*Hungarian herding dogs*), where Trial 1 had longer latency than Trial 2–3 (Figure 5b); in Group 6 (*German Shepherd Dog, Hovawart*), where each trial differed from the other (Figure 5d); and Group 10 (*sight hounds and English herding dogs*), where again, each trial differed from the other (Figure 4g).

**FIGURE 6:**
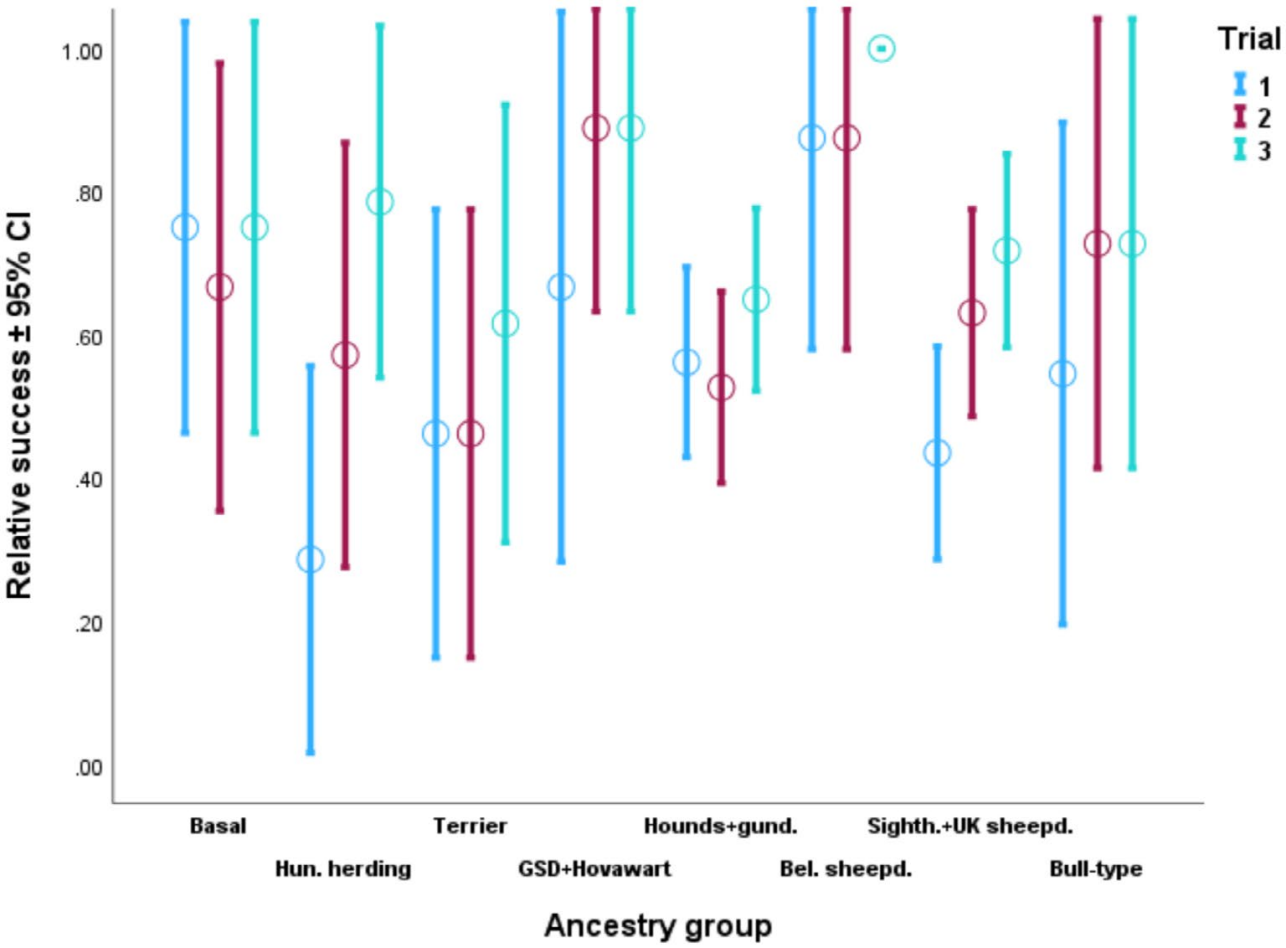
Success rate of detours in each ancestry group, across the trials. Whiskers = 95% confidence interval (CI); circles = mean.

We compared the dogs' across‐trial success rates among the clades. While the confounding factors did not have a significant effect (breed function: *χ* ^2^_(1)_ = 1.447; *p* = 0.229; demonstration: *χ* ^2^_(2)_ = 0.235; *p* = 0.889; keeping: *χ* ^2^_(1)_ = 0.057; *p* = 0.811; training: *χ* ^2^_(5)_ = 1.147; *p* = 0.950), ancestry groups (*χ* ^2^_(7)_ = 495.529; *p* < 0.001) and trials (*χ* ^2^_(2)_ = 774.825; *p* < 0.001) did. Additionally, we found a strong significant interaction effect between trials and ancestry groups (*χ* ^2^_(11)_ = 271.581; *p* < 0.001). On Figure 6 we can see that Group 6 (*German Shepherd Dog, Hovawart*), and Group 9 (*Briard and Belgian sheepdogs*) had rather high success rates in all three trials. While success rates showed strong improvement across the trials in Group 3 (*Hungarian herding dogs*) and Group 10 (*Sighthounds and English herding dogs*), the opposite was true for Group 1 (*basal breeds*) and Group 8 (*gundogs and hounds*), where the success rate did not differ between the trials.

We analysed whether the relative duration of watching the demonstrator (only in the case of Trials 2–3, and without the dogs who participated in no‐demo control groups) was in association with the ancestry group, breed function, keeping condition, training level, trial (repeated factor) and with the success in that trial (Table 5). We found that ancestry group, demonstration and dogs' success had a significant association with watching the demonstrator.

**TABLE 5:** Results of the GEE analysis in the case of relative duration of watching the demonstrator.

Independent variable	Wald *χ* ^2^	df	*p*
**Ancestry group**	**45.565**	**7**	**< 0.001**
Breed function	1.895	1	0.169
**Demonstration**	**4.039**	**2**	**0.044**
Keeping	0.000	1	0.983
Training	9.990	5	0.076
**Success**	**4.368**	**1**	**0.037**
Trial	2.153	1	0.142

*Note:* Significant associations are highlighted with bold typeset.

**FIGURE 7:**
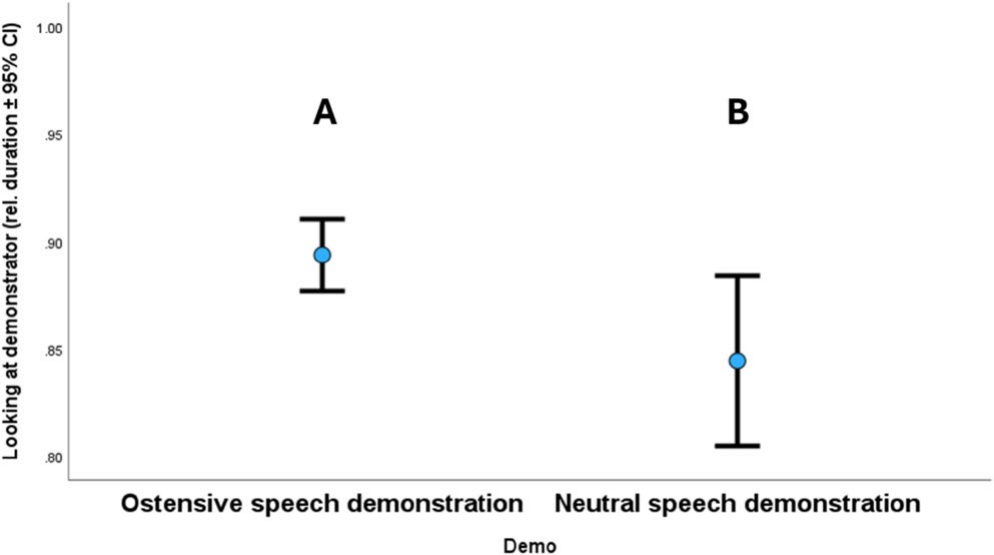
Relative duration of watching the demonstration in the case of ostensive and neutral‐speech demonstrations. Different letters mark significant difference between the groups. Whiskers = 95% confidence interval (CI). Circle, mean.

**FIGURE 8:**
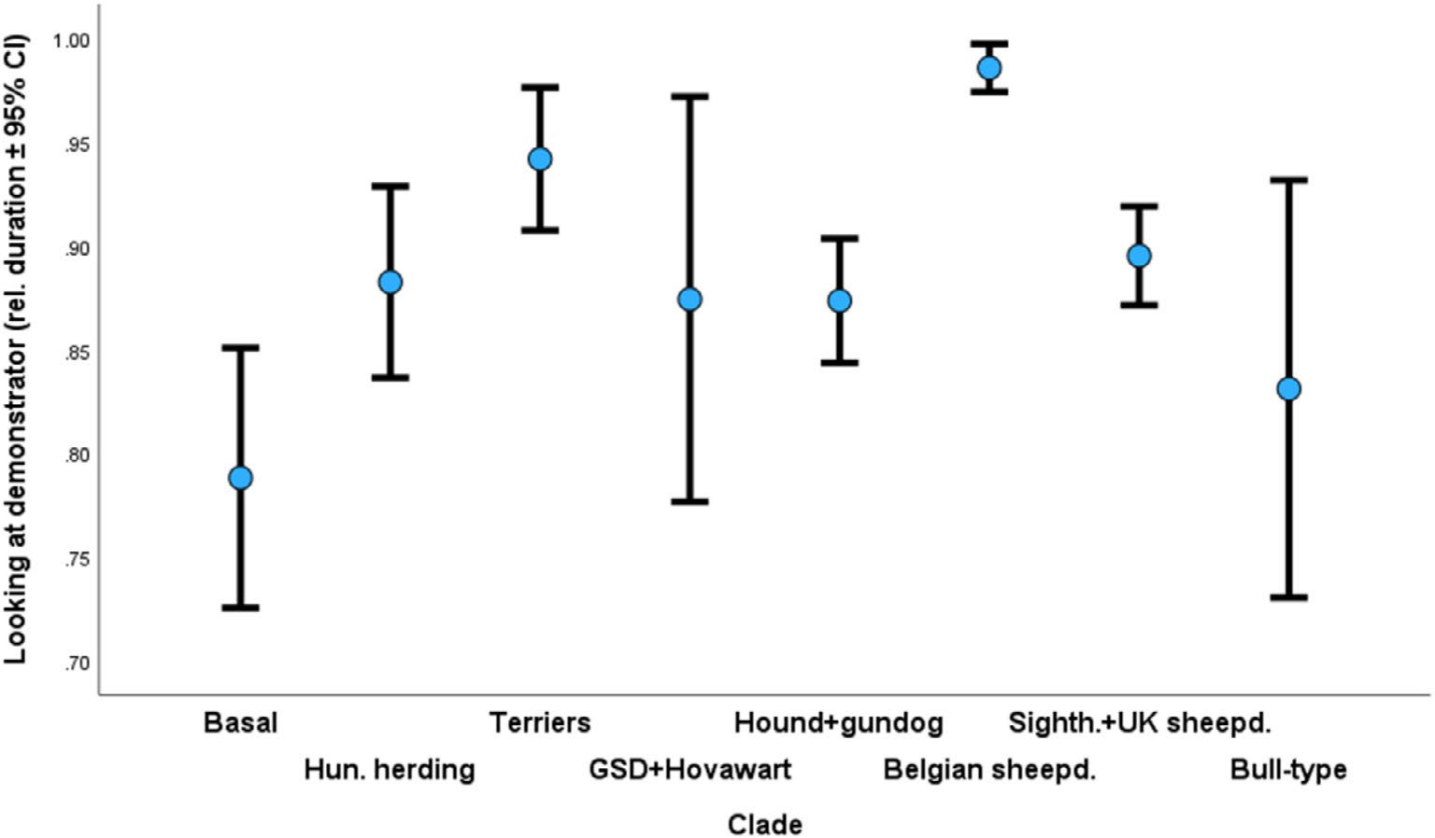
Relative duration of watching the demonstration in the case of the ancestry groups. Group 9 (Briard, Belgian sheepdogs) watched the demonstration significantly longer than any other group. Group 1 (basal breeds) watched the demonstrator the shortest.

Dogs who watched the demonstration longer were more successful in detouring the fence. Dogs made more successful detours if they received demonstrations with ostensive speech by the demonstrator compared to the dogs who observed neutral‐speech demonstrations (Figure 7). Finally, regarding the ancestry groups, Group 9 (*Briard, Belgian sheepdogs*) significantly looked the longest at the demonstrator, while dogs in Group 1 (*ancient breeds*) watched the demonstration less than most other groups (Figure 8).

## 4 Discussion

Based on a large, unified database we analysed the behaviour of purebred working dog breeds from three studies that were based on the detour paradigm to test potential associations with the genetic relatedness of the breeds. We found that the detour latencies in the first trial were similar across ancestry groups. Consequently, the detour task was uniformly difficult for each dog, which was expected based on our previous findings related to functional breed selection (Dobos and Pongrácz 2023, 2024). This result shows that the ancient (basal) breeds do not have higher individual problem‐solving skills in the detour task than the more derived breeds do. This may come as a surprise when one takes into consideration the ancient breeds' behavioural features, which otherwise can be important for successful problem‐solving. Inhibitory control is a fundamental asset for successfully performing the detour task (Kabadayi et al. 2018), because participants must refrain from their direct approach attempts to be able to depart from the unreachable reward. Based on comparative results on socialized wolves and group‐kept mongrel dogs, wolves had higher inhibitory control than dogs in the detour task (Marshall‐Pescini et al. 2015), thus we could also expect higher inhibitory control (and consequently, better performance) in the detour task from the “ancient” breeds. Additionally, the ancient breeds' general inefficiency in the detour task that was detected in our study, contradicts the expectation that the ancient breeds' higher persistence found in other scenarios (e.g., “unsolvable task”: Maglieri et al. 2019), would also result in higher success rates in a spatial task. At the same time, our results about the dogs' relative looking time at the demonstration aligned well with earlier studies, where “ancient” breeds have been found to gaze less at nearby humans than the more modern breeds did (e.g., Passalacqua et al. 2011; Konno et al. 2016). The generally pronounced inattentiveness of ancient breeds towards human activity could be an additional factor why these dogs had lower performance in the spatial problem‐solving task.

In Trials 2 and 3, the latency of reaching the reward showed strong association with the dog breeds' ancestry‐group assignment. Therefore, we can assume that the distance from the common ancestor could influence how dogs utilize human demonstration as a general source of social learning, because the type of demonstration (ostensive attention eliciting vs. neutral speech during the detour), did not affect the results. As we hypothesized, some of the more derived clades where the dog breeds were probably more directly selected for interacting with humans (Nagasawa et al. 2017), their latencies improved more. We found that those ancestry groups were the most effective in Trials 2–3 (i.e., observing the demonstration) which also comprise some of the most popular utility breeds (German Shepherd Dogs, Belgian sheepdogs). Apart from Hovawarts, the dog breeds of these ancestry groups belong to the cooperative type; thus, one could assume that the superiority of these ancestry groups in social learning was confounded by functional breed selection (Pongrácz and Dobos 2025). Our other results seem to confirm this assumption, as we found that cooperative dogs improved their detour latencies trial‐by‐trial more than the independent dog breeds when we analysed the whole sample together. However, other ancestry groups also incorporate numerous cooperative breeds (e.g., Group 3—Hungarian herding dogs; Group 8—gundogs; and Group 10—English herding dogs), and these groups did not show high performance in the trials where social learning could help their attempts to detour. While the independent working breeds, which are also included to Group 8 (hounds) and Group 10 (sighthounds) could explain the lack of improvement of detour latencies in these groups, Group 3 only comprises highly cooperative herding dogs that also showed rather long detour latencies. Therefore, the previous results (Dobos and Pongrácz 2023, 2024) on the association between functional breed selection and social learning cannot completely explain our results in the current analysis.

Within‐subject latency improvement across the trials showed that there were some ancestry groups (e.g., the basal breeds, terriers, and bull‐type/Molossoid breeds) that definitely did not improve their detour speed even after observing two demonstrations at the beginning of Trials 2 and 3. Besides the superb performance of the GSD/Hovawart group, two additional groups, where the herding breeds (Hungarian and English breeds) were clustered, learned most effectively from the demonstrator. This could be the result of the cooperative and therefore dependent nature of these breeds (Pongrácz and Lugosi 2024), which manifests itself in a more willing reliance on human‐given communicative and behavioural cues (Gácsi et al. 2009; Bognár et al. 2021; Dobos and Pongrácz 2024). Dependence on human assistance (i.e., demonstration) in herding breeds was also indirectly supported by their low performance in the first trial. On the other hand, while the group with the English herding breeds performed well, the group with the likewise cooperative gundogs did not, therefore we found an indication that cooperativity is not the only underlying factor of high performance in dogs that can learn from a human demonstrator. Ancestry groups 8 and 10 both contain closely related dog breeds which are at the same time represent cooperative and independently working dogs by their function. Alongside the cooperative sheepdogs, Group 10 contains independently working sight hounds as well, which all belong to the original “Clade T' on Parker and colleagues” cladogram (2017). Group 10 comprises three closely related clades (O, P, and Q) from the Parker et al. (2017) cladogram, including the independently working scent hounds and Dachshunds besides the cooperative gundogs. This suggests that within the independent functional breed type, the exact nature of work that the breeds have been selected for may still influence the dogs' social learning capacity. As a result, scent hounds and/or Dachshunds might be particularly insensitive to human demonstrations, unlike the sight hounds. The latter, together with the closely related English herding breeds, performed well in the social learning task.

Furthermore, the type of work these dogs were bred for can have similarities even if the breeds otherwise belong to either the cooperative or independent type. For example, English herding dogs and sight hounds from Group 10 perform their task mainly by using their vision, and they all stalk and chase the “prey animals” with only one main difference which concerns the end‐phase of the “hunt”.

Regarding the duration of watching the demonstration, we found that dogs were more successful when they watched the demonstration for a longer period. These results confirm our earlier findings, where we found a positive association between the dogs' success rate and their observation of the demonstrator (Dobos and Pongrácz 2024, 2025). However, while it was found earlier that mostly the cooperative working dog breeds preferred watching the human demonstrator (Dobos and Pongrácz 2024), here the breed function alone as a factor did not associate with the relative duration of watching the demonstrator. On the other hand, the more derived ancestry Group 9, which includes the Briard and Belgian sheepdogs, significantly looked the longest at the demonstrator, while the ancient breeds watched the demonstration less than most other groups. Interestingly, other groups with high success rates and significantly improving detour latencies, showed only medium‐level interest towards the demonstrator. This result indicates that ancestry groups may show strong differences in their interest towards human activity, which is partially independent from the functional selection type of the breeds that they represent.

Overall, there is a potential overlap between the effects of ancestry and functional breed selection. Functional selection resulted in a wide variety of breeds, originating from often very distant genetic clades, although, most cooperative breeds can be found in the more derived ancestry clades (Parker et al. 2017). However, our results suggest that the performance of different dog breeds cannot be completely explained by functional breed selection. We found only one significant effect of breed function, in the case of the analysis of reward latencies on the whole sample, where cooperative dogs made faster detours than the independent breeds. As earlier we did not find difference between the performance of the two functional breed types when the dogs did not get demonstration in the control trials (Dobos and Pongrácz 2023, 2024), this effect now can be an indication of the different effect of demonstrations on the two breed types. Otherwise, in the case of the more detailed analyses which now included ancestry groups as well, there were groups that performed the detour task in contrast with the expectation that we would have based on their functional background, such as groups including cooperative breeds that did not show high performance in social learning (e.g., Group 8 with numerous gundogs, or Group 11 with utility breeds such as the Boxer and Rottweiler). Curiously, the group that performed the best, included both cooperative and independent working dogs (Group 6 with German Shepherd Dogs and Hovawarts), meanwhile the second‐best performing group (Group 9 with the Briard and Belgian sheepdogs) comprised cooperative breed only. One could assume a potential super‐position between functional selection and ancestry‐based effects. The derived breeds that underwent strong initial artificial selection, followed by a more recent functional selection regarding other traits they were selected for such as cooperativeness and/or dependence, could become the most attentive to human behaviour. Consequently, these groups provide the most useful utility breeds for service tasks such as police work, military, and search‐and‐rescue, where biddability, cooperativeness and persistence are important traits (e.g., Helton 2009; D'Aniello et al. 2015; Lazarowski et al. 2019; Morrill et al. 2022).

In our study we used a wide selection of working dog breeds that extensively cover the Parker‐cladogram from the very ancient to the modern breeds (2017). The detour task coupled with social learning is a biologically relevant scenario which includes both individual and social problem‐solving elements. Unlike the narrower sampling in other studies (e.g., Nagasawa et al. 2017), and the comparison among individual dog breeds' performance in studies that used convenience sampling methods (e.g., Junttila et al. 2022), in this study we had the opportunity to take into consideration both the overarching effect of ancestry and function. With the help of a broader analysis, our earlier findings (Dobos and Pongrácz 2023, 2024) about the social learning ability of working dog breeds can now be placed into a different point of view. The previously found superior performance of the cooperative functional group in the social learning context could be the average of highly performing utility and herding breeds and the moderately successful gundog breeds. Nevertheless, in the current analysis, the attentiveness to the demonstration did not show a clear effect of ancestry, indicating that functional breed selection can be the dominant underlying factor for the sensitivity to ostensive and neutral human speech.

Probably the most relevant limitation of our study was the uneven sample size among our ancestry groups. Furthermore, some of the groups consisted of breeds belonging to one clade while the others were the result of clustering multiple clades. In the case of Group 6 (GSD and Hovawart) and 9 (Briard and Belgian sheepdogs) the low sample size and rather homogenous breed composition may allow that random individual factors bias the results. However, keeping and training did not affect the results of this study, which lessens the chance of such confounders being responsible for the results. Another limitation of the study is the use of only working dog breeds, therefore we had to make “ancestry groups” by merging two or more closely related clades from the original Parker‐cladogram, and not all clades were represented in the study properly. Even after the clustering of the clades, some “ancestry groups” had relatively low sample sizes. However, we were often able to find significant results even in the case of these groups, which may indicate a robust effect of clades/groups. Moreover, some dog breeds can potentially have “working and show” lines which could represent another underlying factor (e.g., Bryant et al. 2018). Our sample came from companion dog owners, thus it is less likely that strictly working line dogs occur in high numbers that would bias the sample. Moreover, if working line dogs had been overrepresented, their specialized training might have influenced performance, however, our findings indicate that training had no effect on either latency or success. Another limitation is that in our analysis there was a smaller proportion of dogs who were tested in the “control version” of the detour test, where they did not receive any demonstration during the three consecutive trials. However, as the demonstration did not have an effect in any of the analyses, we can conclude that the dogs who were originally tested in the “Control” (no demo) condition did not bias the results, as they were distributed evenly across the ancestry groups.

## 5 Conclusion

Ancestry is another ecologically valid grouping variable if the genetic relationship‐based behavioural analysis is carried out on a large number of breeds (e.g., Morrill et al. 2022), or if sufficiently sampled breed clusters are compared (e.g., Hansen Wheat et al. 2019). An ancestry‐based method is especially suitable for the investigation of between‐breed differences and has gained popularity in recent empirical research (e.g., dogs following human pointing gestures; Dorey et al. 2009). However, when drawing conclusions based on genetic relatedness, besides the effects of domestication and early genetic‐clade formation based on certain behavioural traits, it is also important to consider the potential effects of more recent artificial selection (Gnanadesikan et al. 2020), as breeds with a similar inclination for dog–human interactivity levels (e.g., cooperative versus independent breeds) can also be found in distant genetic clades (Parker et al. 2017; Morrill et al. 2022; Dobos and Pongrácz 2023). Moreover, this method is less suitable for analysing behavioural differences between closely related breeds (Dorey et al. 2009).

In conclusion, our new analysis showed that the more ancient breeds that underwent weaker directional selection, have lower success in a social learning task, while the more recent, derived breeds, especially utility dogs, could learn from a human demonstration more easily. However, the attentiveness to the human demonstrator was not affected profoundly by the ancestry‐groups, supporting the idea that the initial directional selection alone, without strong functional purpose, was not enough to form different levels of human‐directed attentiveness. Ancestry is an ecologically valid grouping variable, but only if sufficiently sampled breed clusters are compared to each other (Pongrácz and Dobos 2025). On the other hand, it is important to pay attention to the potential overlap between ancestry and functional breed selection and design studies that can control one or the other if we want to draw biologically relevant conclusions.

## Ethics Statement

Each of the earlier studies, which provided the data to our current research, were conducted in accordance with the Hungarian regulations on animal experimentation and guidelines for use of animals in research, as outlined by the Association for the Study of Animal Behaviour (ASAB). This fully non‐invasive experimentation methodology was reviewed and approved by the Animal Welfare Committee of the University (Certificate number ELTE‐AWC‐013/2023). Companion dogs were always tested in the presence of their owners. Tests were reward‐based and fully non‐invasive, however, we informed the owners that they could interrupt or stop the experiments any time they felt that their dog experienced high levels of stress.

## Consent

All authors approved and consented to the publication.

## Conflicts of Interest

The authors declare no conflicts of interest.

## Supporting information


**Data S1:** Raw_data_Ancestry_detour_FINAL.

## Data Availability

The raw data are accessible through Mendeley Data (Pongrácz and Dobos (2025), “Ancestry_detour_dogs”, Mendeley Data, V2, doi: 10.17632/yyg9cw67hk.2).
